# Genomic and Transcriptomic Comparison Between Invasive Non-Typhoidal *Salmonella* and Non-Invasive Isolates

**DOI:** 10.3390/microorganisms12112288

**Published:** 2024-11-11

**Authors:** Tongyao Shang, Qiuli Chen, Weina Shi, Yue Wang, Ye Feng

**Affiliations:** 1 Sir Run Run Shaw Hospital, Zhejiang University School of Medicine, Hangzhou 310020, China; 2 Institute of Translational Medicine, Zhejiang University School of Medicine, Hangzhou 310029, China; 3 Women’s Hospital, Zhejiang University School of Medicine, Hangzhou 310006, China

**Keywords:** antimicrobial resistance, genome, invasiveness, pseudogene, *Salmonella*, transcriptome

## Abstract

Invasive non-typhoidal *Salmonella* (iNTS) poses a significant threat to global public health. *Salmonella enterica* Enteritidis and Typhimurium are the primary serovars responsible for both invasive diseases and gastroenteritis. This study aims to investigate the genomic and transcriptomic differences between isolates associated with these contrasting clinical presentations. We retrieved genomes of *Salmonella* Enteritidis and *Salmonella* Typhimurium from Enterobase, utilizing blood and stool isolates as representatives for iNTS and non-iNTS, respectively. An indistinguishable phylogenetic relationship was revealed between the blood and stool isolates for both serovars. Few genes were specifically identified in iNTS. Random forest and principal coordinates analysis permitted moderate discrimination between the two sources of isolates based on overall genome content. Notably, the blood isolates of *Salmonella* Typhimurium displayed an elevated level of antimicrobial resistance and genome degradation compared to stool isolates. Meanwhile, transcriptome sequencing identified few genes that were differentially expressed between blood and stool isolates. Hierarchical clustering and principal component analysis did not effectively differentiate the expression profile of iNTS from non-iNTS. In summary, few genes could serve as reliable biomarkers to distinguish iNTS and non-iNTS at either the genomic or transcriptomic level. Nevertheless, iNTS has indeed accumulated subtle genomic differences from non-iNTS, which might contribute to invasiveness.

## 1. Introduction

Among the nearly 2000 serovars of *Salmonella enterica*, the typhoidal serovars, such as *Salmonella* Typhi and *Salmonella* Paratyphi A, have a limited host range within humans and cause systemic infections. In contrast, non-typhoidal *Salmonella* (NTS) serovars can usually infect a wide variety of animals, including mammals, birds, and reptiles, and are primarily associated with self-limiting gastroenteritis. However, NTS can also cause invasive infections occasionally, such as bacteremia, which carries a mortality rate of up to 14.7% [1]. The risk of invasive non-typhoidal *Salmonella* (iNTS) infections is closely linked to the host’s health and immune status, with children, the elderly, and immunocompromised individuals identified as being at elevated risk [2]. The pathogenicity of iNTS is influenced not only by host factors but also by specific bacterial characteristics. Globally, *S. enterica* serovar Typhimurium and Enteritidis are responsible for the majority of iNTS cases [2]. Notably, the sequence type (ST) 313 of *Salmonella* Typhimurium predominates in iNTS cases in Africa but is rarely encountered on other continents [3,4]. Other serovars, including Heidelberg, Choleraesuis, Dublin, and Panama, account for only a small fraction of salmonellosis cases but are recognized for their high invasive potential [5,6].

Although iNTS is well-recognized as a clinical concept, the current understanding of its molecular mechanisms is limited, with much of the research focused on ST313. Studies have identified the genomic, transcriptomic, and phenotypic differences related to cellular invasion and survival between ST313 and non-ST313 *Salmonella* Typhimurium [3,7,8,9]. A few studies have also explored the virulence factors contributing to the highly invasive serovars [10]. However, several fundamental questions about iNTS remain unresolved. Firstly, *Salmonella* Typhimurium and Enteritidis are not only primary pathogens in iNTS infection but are also major causes of gastroenteritis. Are there intrinsic biological differences between isolates that cause iNTS and those causing gastroenteritis? Secondly, do different invasive serovars share common biological features that enhance their invasiveness? Finally, is iNTS infection more dependent on the pathogen’s characteristics or host factors? Through genome and transcriptome comparisons, we aim to elucidate the molecular mechanisms driving iNTS invasiveness and identify genetic features that may differentiate iNTS from non-iNTS isolates.

## 2. Materials and Methods

### 2.1. Data Collection

Genome sequences of *Salmonella* isolates were downloaded from Enterobase v1.2.0 [11], along with core-genome multilocus sequence typing (cgMLST) and whole-genome multilocus sequence typing (wgMLST) profiles. Metadata indicating whether the isolates were collected from stool or blood were also downloaded from Enterobase.

Transcriptomic data were generated in the present study. Clinical *Salmonella* isolates were obtained from the clinical lab at Sir Run Run Shaw Hospital, Hangzhou, China. The isolates were serotyped with a multiplex PCR-based method [12]. The bacteria were cultured overnight in LB medium and then were transferred into fresh LB medium at a 1:100 dilution. When the cultures reached the early stationary phase (OD_600_ 2.0), the bacteria were harvested, and total RNA was extracted with RNeasy Kit (QIAGEN, Germantown, MD, USA). Ribosomal RNA was removed by RiboZero kit (Illumina, San Diego, CA, USA). Fragmentation, cDNA synthesis, end repair, and the ligation of the Illumina-indexed adaptors were performed according to Illumina’s protocol. The library was then sequenced on an Illumina Nova-seq 6000 platform, generating 150 bp paired-end reads with a minimum sequencing throughput of 2 Gb.

### 2.2. Data Analysis

#### 2.2.1. Genome Comparison

In silico seven-gene multilocus sequence typing (MLST) was performed on the *Salmonella* genomes using the BacWGSTdb service [13]. Based on the predicted sequence type, isolates with incorrect serovar information in the metadata were removed. To minimize potential statistical bias from isolate redundancy, the pairwise differences in cgMLST loci between isolates were counted. Isolates differing by fewer than five cgMLST loci were considered redundant; only one isolate from each group of redundant isolates was retained. If a stool isolate and a blood isolate showed fewer than five cgMLST loci differences, they were assumed to belong to the same case, and both the stool and blood isolates were excluded from further analysis. GrapeTree software v1.5.0 was used to construct phylogenetic relationships [14], using the cgMLST profile as input.

Four types of genetic features were analyzed in this study, including antimicrobial resistance (AMR) genes, pseudogenes, pseudo-operons, and protein-coding genes (CDS). The AMR genes were identified in the genomes using the ResFinder database v4.6.0 [15]. To detect pseudogenes, the genome of *Salmonella* Typhimurium strain 14028S (accession no. NC_016856) was used as the reference. Protein sequences from the reference genome were searched against the query genomes using the tblastn program in NCBI blast v2.7.1. The nucleotide sequences of the orthologous genes in the query genome were obtained and further compared with the reference protein sequence using GeneWise software v2.4.1 [16]. The query orthologous genes were considered pseudogenes if they contained frameshifts or nonsense mutations. The operon information was obtained from OperomeDB [17]. An operon was considered a pseudo-operon if any gene within it was a pseudogene. The information of whether a CDS was present in an isolate’s genome was retrieved from the wgMLST profile: if a CDS was assigned an allele number in the query genome, it was considered present in that isolate. 

All features described above were organized into a binary (0/1) matrix according to their presence/absence in the query genomes. A chi-square test was performed for each feature, and the *p*-values were adjusted using the Benjamani–Hochberg (BH) method. The random forest algorithm, implemented by using the randomForest R package v4.7, was used to distinguish stool and blood isolates. The datasets were partitioned into training (70%) and testing (30%) sets using caret’s createDataPartition function. Model performance, including the area under the receiver operating characteristic curve (AUROC), was assessed using the pROC R package v 1.18.5 [18]. The datasets also underwent principal coordinates analysis (PCoA) and Adonis analysis, which were calculated using the vegan R package v2.6.2 [19].

#### 2.2.2. Transcriptome Comparison

Low-quality bases and adapter sequences in the raw transcriptome sequencing reads were trimmed using Trimmomatic v0.39 [20]. The cleaned reads were aligned to the reference genome using HISAT2 v2.2.1 with default parameters [21]. Due to the genomic differences between the serovars, different reference genomes were used based on each isolate’s serovar. The reference genomes for the serovar Enteritidis, Typhimurium, Choleraesuis, Dublin, Heidelberg, Panama, Paratyphi A, and Typhi were NC_011294, NC_016856, NC_006905, CP019179, CP016576, CP012346, NC_006511, and NC_004631, respectively. OrthoFinder v2.5.5 [22] was used to construct the orthologous relationship between the genes of these serovars, resulting in 3134 genes that were conserved across the above serovars. To minimize the effect of serovar-specific genes upon the transcriptome analysis, the annotation GTF file contained the 3134 genes only. Aligned reads were quantified using FeatureCounts v2.0.2 [23], which generated a final table of read count for the 3134 conserved genes. DESeq2 R package v1.38.3 was used for identification of differentially expressed genes [24]. Principal component analysis (PCA) was conducted to reduce the dimensionality of the dataset by using the PCA function from the FactoMineR R package v2.11. Hierarchical clustering, followed by heatmap generation, was performed with the pheatmap R package v1.0.12.

## 3. Results

**Figure 1:**
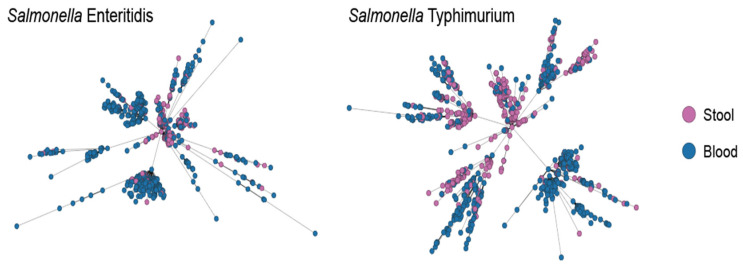
The phylogenetic relationship between blood and stool isolates for *Salmonella* Enteritidis and Typhimurium. The phylogenetic trees were built based on cgMLST profile.

Here, we took the isolates derived from human stool and blood as representatives of gastroenteritis and iNTS, respectively. Phylogenetic analysis based on cgMLST revealed that isolates from the two sources could not be distinguished in either *Salmonella* Typhimurium or *Salmonella* Enteritidis (Figure 1).

**Figure 2:**
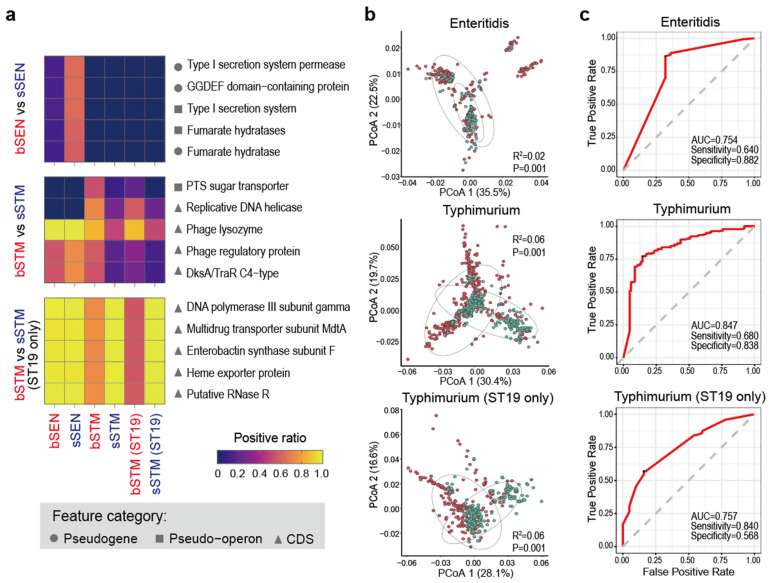
Comparison of gene content between blood and stool isolates for *Salmonella* Enteritidis and Typhimurium. (**a**) Top 5 features (i.e., with the smallest *p*-value) that are differentially distributed in blood and stool isolates. The heatmaps show the positive ratio of the feature in isolates. As this panel illustrates, even these Top 5 features do not achieve a nearly 0/1 difference in positive ratio in the pairwise comparison. bSTM, blood-sourced Typhimurium; sSTM, stool-sourced Typhimurium; bSEN, blood-sourced Enteritidis; sSEN, stool-sourced Enteritidis. (**b**) Principal coordinates analysis and Adonis analysis performed between stool and blood isolates. Red and green dots represent blood and stool isolates, respectively. (**c**) Receiver operating characteristic curves produced by the random forest classification between stool and blood isolates.

**Figure 3:**
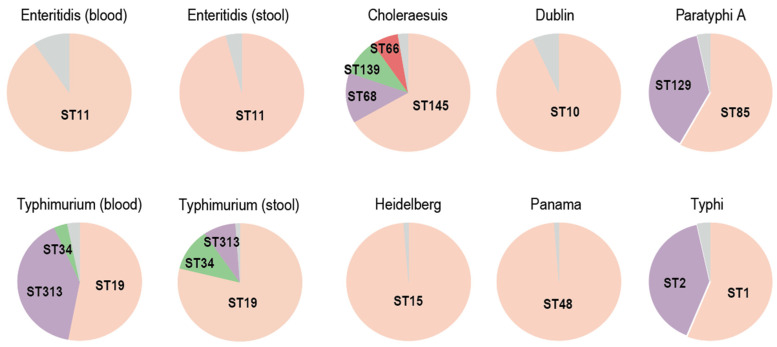
Distribution of sequence type (ST) for each serovar according to the number of the analyzed isolates. The gray color indicates other sequence types.

**Figure 4:**
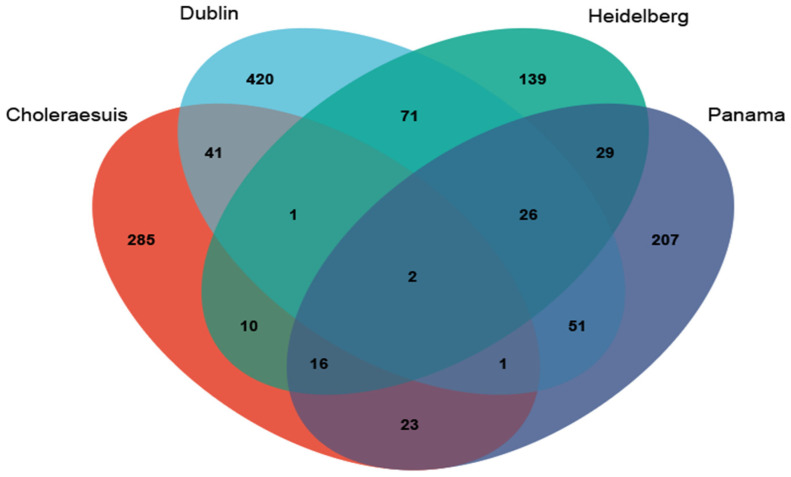
Venn diagram showing the overlap of genetic features enriched in different invasive serovars. Each of these invasive serovars is compared against the non-iNTS isolates (i.e., combination of the stool Typhimurium isolates and the stool Enteritidis isolates). A small number of features are shown to be commonly enriched in these different invasive serovars (see detail lists of the overlapped features in Supplementary Materials Table S1).

Next, we characterized iNTS using genetic features, including AMR genes, pseudogenes, pseudo-operons, and protein-coding sequences (CDSs). Few features effectively distinguished the stool and blood isolates in *Salmonella* Enteritidis (Figure 2a and Supplementary Materials Figure S1). The differences between the stool and blood *Salmonella* Typhimurium isolates were more pronounced, largely due to the significantly higher proportion of ST313 in the blood isolates (Figure 3). When ST313 was excluded, far fewer differential features were identified between stool and blood isolates, with no overlap in the differences observed in *Salmonella* Enteritidis (Figure 2a). We also identified genes enriched in invasive serovars, including *Salmonella* Choleraesuis, Dublin, Heidelberg, and Panama. Only a small number of genes were shared among these serovars (Figure 4 and Supplementary Materials Table S1), indicating that their invasiveness was not driven by the same genes. These genes primarily function in encoding the cell membrane, fimbria, and carbon metabolism.

**Figure 5:**
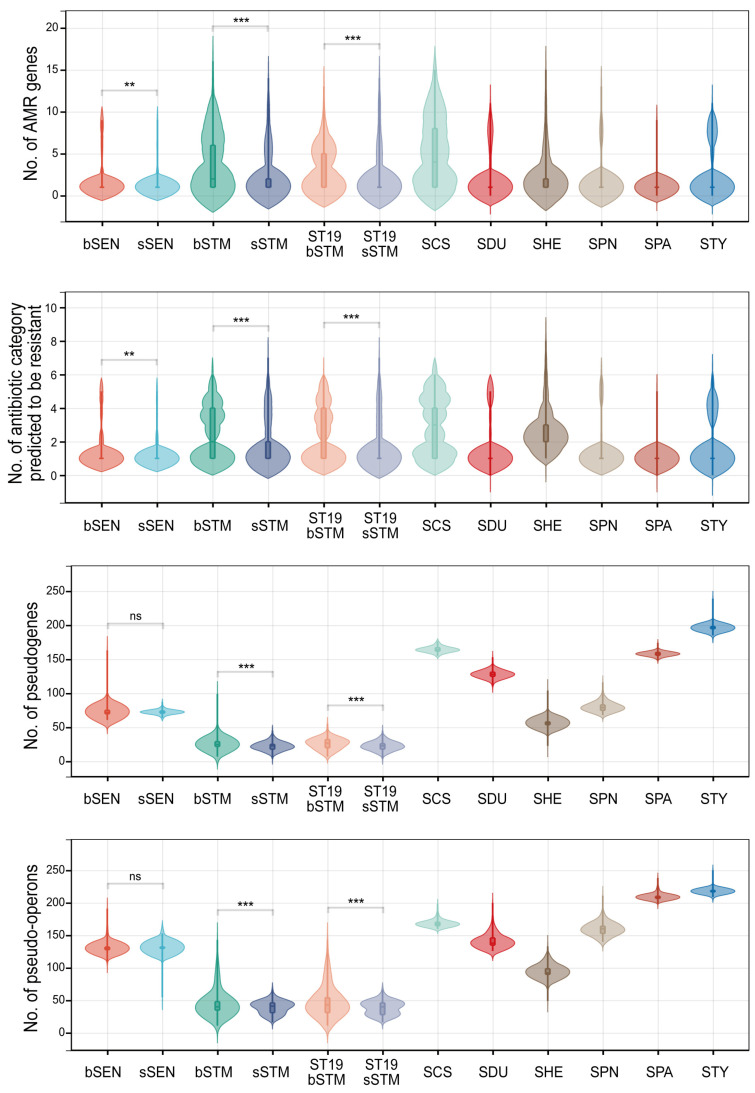
Number of genetic features accumulated in *Salmonella* isolates with different sources. Student’s *t*-test is performed to compare between bSEN and sSEN and between bSTM and sSTM. **, *p* < 0.01; ***, *p* < 0.001; ns, not significant. bSTM, blood-sourced *Salmonella* Typhimurium; sSTM, stool-sourced *Salmonella* Typhimurium; bSEN, blood-sourced *Salmonella* Enteritidis; sSEN, stool-sourced *Salmonella* Enteritidis; SCS, *Salmonella* Choleraesuis; SDU, *Salmonella* Dublin; SHE, *Salmonella* Heidelberg; SPN, *Salmonella* Panama; SPA, *Salmonella* Paratyphi A; STY, *Salmonella* Typhi.

The comparison of the total number of features revealed that the blood isolates of both *Salmonella* Typhimurium and Enteritidis contained a greater number of AMR genes than the stool isolates (Figure 5). Even after excluding ST313, the blood isolates of *Salmonella* Typhimurium displayed a higher prevalence of pseudogenes and pseudo-operons compared to the stool isolates, although this trend was not observed in *Salmonella* Enteritidis. Significant pseudogenization was noted in *Salmonella* Choleraesuis and Dublin, but not in *Salmonella* Heidelberg and Panama (Figure 5). Using all features, principal coordinates analysis weakly but significantly distinguished blood isolates from stool isolates in both *Salmonella* Typhimurium and Enteritidis (Adonis test, *p* = 0.001; Figure 2b). Similarly, random forest analysis also made the discrimination, achieving an AUC greater than 0.7 (Figure 2c).

**Figure 6:**
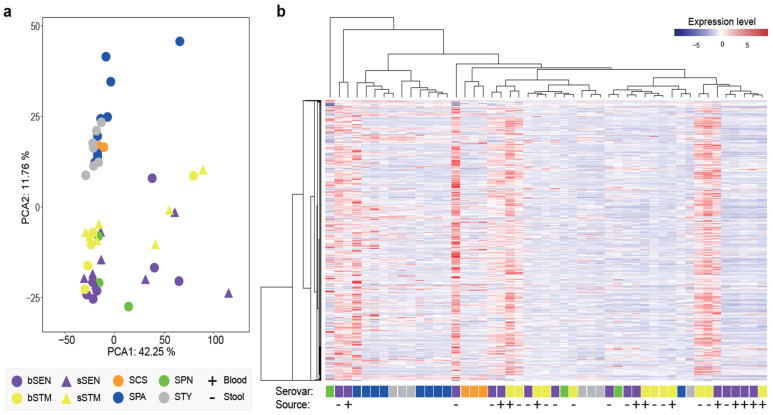
Transcriptomic comparison between blood and stool isolates for *Salmonella* Enteritidis and Typhimurium. The *Salmonella* isolates are cultured in LB medium. (**a**) Principal component analysis of the expression profile. (**b**) Hierarchical clustering based on the expression profile. bSTM, blood-sourced *Salmonella* Typhimurium; sSTM, stool-sourced *Salmonella* Typhimurium; bSEN, blood-sourced *Salmonella* Enteritidis; sSEN, stool-sourced *Salmonella* Enteritidis; SCS, *Salmonella* Choleraesuis; SDU, *Salmonella* Dublin; SHE, *Salmonella* Heidelberg; SPN, *Salmonella* Panama; SPA, *Salmonella* Paratyphi A; STY, *Salmonella* Typhi.

Lastly, we sequenced the transcriptomes of *Salmonella* isolates that were cultured in LB medium. To minimize the impact of serovar-specific genes on the global expression profile, our analysis focused exclusively on genes conserved across the entire *S. enterica* species. Both hierarchical clustering and principal component analysis revealed that isolates within the same serovar exhibited greater similarity to each other (Figure 6). Additionally, the expression profiles of *Salmonella* Typhimurium and *Salmonella* Enteritidis isolates were more variable within each serovar compared to those of *Salmonella* Choleraesuis, *Salmonella* Paratyphi A, and *Salmonella* Typhi. Notably, the stool and blood isolates could not be distinguished from one another, with few genes showing differential expression between them (Figure 6).

## 4. Discussion

In this study, we conducted a comprehensive genomic comparison between stool-sourced and blood-sourced isolates and discovered that blood isolates contained a higher number of AMR genes, thereby exhibiting a greater likelihood of multidrug resistance traits. Previous literature has indicated that iNTS isolates are more resistant to antimicrobials, including first-line drugs such as ciprofloxacin and ceftriaxone [2,25], although some reports have presented contrasting findings [26,27]. This enhanced resistance implies that iNTS can lead to more severe disease and complicates treatment strategies. However, we did not identify any specific AMR genes that were particularly enriched in iNTS compared to non-iNTS isolates. Thus, this elevated resistance does not appear to be mediated by specific plasmids but rather suggests that AMR contributes to invasiveness and/or that the iNTS isolates may have undergone stronger selective pressure from the antimicrobials.

Previous literature has reported extensive genome degradation in ST313 [3,4]. Compared to ST19, ST313 isolates exhibit numerous pseudogenes with functions related to transcriptional regulation, metabolism, and transport. Here, we found that even when excluding ST313, the blood ST19 isolates still contained a significantly larger number of pseudogenes compared to the stool ST19 isolates. This increase was not limited to specific pseudogenes or operons. Therefore, invasiveness is unlikely to result from the deliberate pseudogenization of certain genes or operons. Instead, the observed pseudogenization may be a consequence of host restriction process, during which the affected genes become dispensable and are no longer subject to strong functional constraints. This hypothesis is further supported by recent findings that iNTS is transmitted in an anthroponotic (human-to-human) manner rather than in a zoonotic manner [28]. Additionally, swine-adapted *Salmonella* Choleraesuis, cattle-adapted *Salmonella* Dublin, and human-restricted *Salmonella* Typhi and *Salmonella* Paratyphi A carry more pseudogenes than the host-generalist *Salmonella* Heidelberg and *Salmonella* Panama.

Similar to AMR genes and pseudogenes, the distribution of CDSs exhibits comparable characteristics. Individual CDSs did not effectively distinguish between stool and blood isolates, reinforcing the notion that these isolates are not phylogenetically distinct. However, the random forest algorithm was able to distinguish the two overall, suggesting that different clinical manifestations may have specific genetic bases at the whole genome scale. It is important to note that such classification should not be directly applied in clinical practice, as its specificity and sensitivity are insufficient to predict whether a *Salmonella* Typhimurium or *Salmonella* Enteritidis isolate will cause iNTS infection or gastroenteritis.

While the majority of previous iNTS studies have focused on specific serovars or ST clades, a few studies have integrated multiple serovars to identify general genetic features contributing to their shared invasive phenotype. Wheeler et al. considered the host-adapted serovars (such as *Salmonella* Dublin and Choleraesuis) to be invasive serovars and proposed assessing invasive potential based on pseudogene composition [29]. Following the same hypothesis, Rakov et al. identified the allelic differences between the so-called invasive serovars and the non-invasive serovars and found that these differences were particularly enriched in virulence genes [30]. A notable similarity between these studies and ours is the significant overlap in the implicated functional categories, highlighting the importance of carbon metabolism, bacterial fimbriae, and type I/III secretion systems in invasiveness. However, what distinguishes our study is our emphasis on comparing blood isolates to stool isolates within the same serovar. Nevertheless, by removing the confounding variable of serovar, we reached the same conclusion that invasiveness may involve multiple genes and/or that different serovars may possess unique mechanisms of invasiveness.

Following the same principle, we also compared the transcriptomes of stool and blood isolates within *Salmonella* Typhimurium and within *Salmonella* Enteritidis. Previous RNA-seq analyses revealed that, compared to ST19, ST313 upregulated the genes associated with cellular invasion and replication, including the effector proteins of the type III secretion system, such as SopD2, SifB, and PipB [31]. However, these transcriptional differences occurred between the two genetically distinct clades rather than between isolates belonging to the same genetic background, but caused different clinical manifestations. Excluding ST313 from this analysis, we found that the expression patterns were clustered by serovar. This serovar-specific expression characteristic may explain the pathogenic traits that are unique to each serovar. For both *Salmonella* Typhimurium and Enteritidis, the stool and blood isolates did not exhibit any differentially expressed genes. While this finding aligns with the genomic observation that iNTS lacks specific genetic markers, it further supports the notion that invasiveness may result from the interplay of multiple genes. However, we cannot rule out the possibility that the culture conditions used in this study may not accurately mimic the in vivo environment of iNTS infections.

## 5. Conclusions

This study represents the first big data-driven, multi-omics comparison for *Salmonella* Typhimurium and *Salmonella* Enteritidis, which are the most prevalent serovars for both gastroenteritis and iNTS infections. This approach addresses two common limitations in previous studies: equating the comparison of iNTS with non-iNTS to comparisons between sequence types, e.g., between ST313 and ST19 *Salmonella* Typhimurium, and generalizing regional (e.g., African) iNTS characteristics to global iNTS traits. We found that iNTS and non-iNTS are phylogenetically indistinct, with few genomic and transcriptomic markers differentiating them. Nevertheless, iNTS exhibits a trend in increasing genome degradation, particularly in genes related to carbon metabolism, bacterial fimbriae, and type I/III secretion systems. While these functions may play crucial roles in invasiveness, the above findings suggest that multiple genes are involved in invasiveness and/or that iNTS infection caused by *Salmonella* Typhimurium and Enteritidis may rely more on host factors than on bacterial factors.

## Data Availability

The raw reads of transcriptome have been deposited in the NCBI Sequence Read Archive (SRA) with the BioProject ID PRJNA1158010.

## Supplementary Materials

The following supporting information can be downloaded at https://www.mdpi.com/article/10.3390/microorganisms12112288/s1: Figure S1: Manhattan plot showing the association of all genetic features with invasiveness; Table S1: Genetic features enriched in invasive *Salmonella* serovars.

## References

[1] Marchello C.S., Birkhold M., Crump J.A. (2022). Complications and mortality of non-typhoidal salmonella invasive disease: A global systematic review and meta-analysis. Lancet Infect. Dis..

[2] Kariuki S., Onsare R.S. (2015). Epidemiology and Genomics of Invasive Nontyphoidal *Salmonella* Infections in Kenya. Clin. Infect. Dis..

[3] Van Puyvelde S., de Block T., Sridhar S., Bawn M., Kingsley R.A., Ingelbeen B., Beale M.A., Barbé B., Jeon H.J., Mbuyi-Kalonji L. (2023). A genomic appraisal of invasive *Salmonella* Typhimurium and associated antibiotic resistance in sub-Saharan Africa. Nat. Commun..

[4] Okoro C.K., Kingsley R.A., Connor T.R., Harris S.R., Parry C.M., Al-Mashhadani M.N., Kariuki S., Msefula C.L., Gordon M.A., de Pinna E. (2012). Intracontinental spread of human invasive *Salmonella* Typhimurium pathovariants in sub-Saharan Africa. Nat. Genet..

[5] Mughini-Gras L., Pijnacker R., Duijster J., Heck M., Wit B., Veldman K., Franz E. (2020). Changing epidemiology of invasive non-typhoid *Salmonella* infection: A nationwide population-based registry study. Clin. Microbiol. Infect..

[6] Fierer J. (2022). Invasive Non-typhoidal *Salmonella* (iNTS) Infections. Clin. Infect. Dis..

[7] Canals R., Hammarlöf D.L., Kröger C., Owen S.V., Fong W.Y., Lacharme-Lora L., Zhu X., Wenner N., Carden S.E., Honeycutt J. (2019). Adding function to the genome of African *Salmonella* Typhimurium ST313 strain D23580. PLoS Biol..

[8] Carden S., Okoro C., Dougan G., Monack D. (2015). Non-typhoidal *Salmonella* Typhimurium ST313 isolates that cause bacteremia in humans stimulate less inflammasome activation than ST19 isolates associated with gastroenteritis. Pathog. Dis..

[9] Ramachandran G., Perkins D.J., Schmidlein P.J., Tulapurkar M.E., Tennant S.M. (2015). Invasive *Salmonella* Typhimurium ST313 with naturally attenuated flagellin elicits reduced inflammation and replicates within macrophages. PLoS Negl. Trop. Dis..

[10] Suez J., Porwollik S., Dagan A., Marzel A., Schorr Y.I., Desai P.T., Agmon V., McClelland M., Rahav G., Gal-Mor O. (2013). Virulence gene profiling and pathogenicity characterization of non-typhoidal *Salmonella* accounted for invasive disease in humans. PLoS ONE.

[11] Zhou Z., Alikhan N.F., Mohamed K., Fan Y., Achtman M. (2020). The EnteroBase user’s guide, with case studies on *Salmonella* transmissions, Yersinia pestis phylogeny, and Escherichia core genomic diversity. Genome Res..

[12] Kim S., Frye J.G., Hu J., Fedorka-Cray P.J., Gautom R., Boyle D.S. (2006). Multiplex PCR-based method for identification of common clinical serotypes of *Salmonella* enterica subsp. enterica. J. Clin. Microbiol..

[13] Feng Y., Zou S., Chen H., Yu Y., Ruan Z. (2021). BacWGSTdb 2.0: A one-stop repository for bacterial whole-genome sequence typing and source tracking. Nucleic Acids Res..

[14] Zhou Z., Alikhan N.F., Sergeant M.J., Luhmann N., Vaz C., Francisco A.P., Carriço J.A., Achtman M. (2018). GrapeTree: Visualization of core genomic relationships among 100,000 bacterial pathogens. Genome Res..

[15] Bortolaia V., Kaas R.S., Ruppe E., Roberts M.C., Schwarz S., Cattoir V., Philippon A., Allesoe R.L., Rebelo A.R., Florensa A.F. (2020). ResFinder 4.0 for predictions of phenotypes from genotypes. J. Antimicrob. Chemother..

[16] Birney E., Clamp M., Durbin R. (2004). GeneWise and Genomewise. Genome Res..

[17] Chetal K., Janga S.C. (2015). OperomeDB: A Database of Condition-Specific Transcription Units in Prokaryotic Genomes. Biomed Res. Int..

[18] Robin X., Turck N., Hainard A., Tiberti N., Lisacek F., Sanchez J.C., Müller M. (2011). pROC: An open-source package for R and S+ to analyze and compare ROC curves. BMC Bioinform..

[19] Dixon P. (2003). VEGAN, a package of R functions for community ecology. J. Veg. Sci..

[20] Bolger A.M., Lohse M., Usadel B. (2014). Trimmomatic: A flexible trimmer for Illumina sequence data. Bioinformatics.

[21] Kim D., Paggi J.M., Park C., Bennett C., Salzberg S.L. (2019). Graph-based genome alignment and genotyping with HISAT2 and HISAT-genotype. Nat. Biotechnol..

[22] Emms D.M., Kelly S. (2019). OrthoFinder: Phylogenetic orthology inference for comparative genomics. Genome Biol..

[23] Liao Y., Smyth G.K., Shi W. (2014). featureCounts: An efficient general purpose program for assigning sequence reads to genomic features. Bioinformatics.

[24] Love M.I., Huber W., Anders S. (2014). Moderated estimation of fold change and dispersion for RNA-seq data with DESeq2. Genome Biol..

[25] Liu F.C., Chang Y.J., Chen C.L., Yang H.P., Lee C.C., Chiu C.H. (2022). Clinical Features, Antimicrobial Resistance, and Serogroups of Nontyphoidal *Salmonella* Isolated From Infants Less Than 3 Months Old in the Recent Decade. Pediatr. Infect. Dis. J..

[26] Phu Huong Lan N., Le Thi Phuong T., Nguyen Huu H., Thuy L., Mather A.E., Park S.E., Marks F., Thwaites G.E., Van Vinh Chau N., Thompson C.N. (2016). Invasive Non-typhoidal *Salmonella* Infections in Asia: Clinical Observations, Disease Outcome and Dominant Serovars from an Infectious Disease Hospital in Vietnam. PLoS Negl. Trop. Dis..

[27] Zou M., He D.M., Xu J., Cheng Q., Ouyang F.Z., Chen L.Y., Chen Q.F., Ke C.W., Ke B.X. (2024). Etiological characterization of invasive non-typhoid *Salmonella* strains in Guangdong Province from 2018 to 2022. Zhonghua Liu Xing Bing Xue Za Zhi.

[28] Koolman L., Prakash R., Diness Y., Msefula C., Nyirenda T.S., Olgemoeller F., Wigley P., Perez-Sepulveda B., Hinton J.C.D., Owen S.V. (2022). Case-control investigation of invasive *Salmonella* disease in Malawi reveals no evidence of environmental or animal transmission of invasive strains, and supports human to human transmission. PLoS Negl. Trop. Dis..

[29] Wheeler N.E., Gardner P.P., Barquist L. (2018). Machine learning identifies signatures of host adaptation in the bacterial pathogen *Salmonella* enterica. PLoS Genet..

[30] Rakov A.V., Mastriani E., Liu S.L., Schifferli D.M. (2019). Association of *Salmonella* virulence factor alleles with intestinal and invasive serovars. BMC Genom..

[31] Martins I.M., Seribelli A.A., Machado Ribeiro T.R., da Silva P., Lustri B.C., Hernandes R.T., Falcão J.P., Moreira C.G. (2023). Invasive non-typhoidal *Salmonella* (iNTS) aminoglycoside-resistant ST313 isolates feature unique pathogenic mechanisms to reach the bloodstream. Infect. Genet. Evol..

