# Supplementary material for: Genomic and Transcriptomic Comparison Between Invasive Non-Typhoidal Salmonella and Non-Invasive Isolates
Source: Microorganisms. 2024 Nov 11;12(11):2288. doi: 10.3390/microorganisms12112288 (PMC11596633; doi:10.3390/microorganisms12112288)
Supplement: Supplementary file 1 [file microorganisms-12-02288-s001.zip › microorganisms-3251636-supplementary.pdf]

Supplementary Materials

I. Supplementary Figure S1

II. Supplementary Table S1

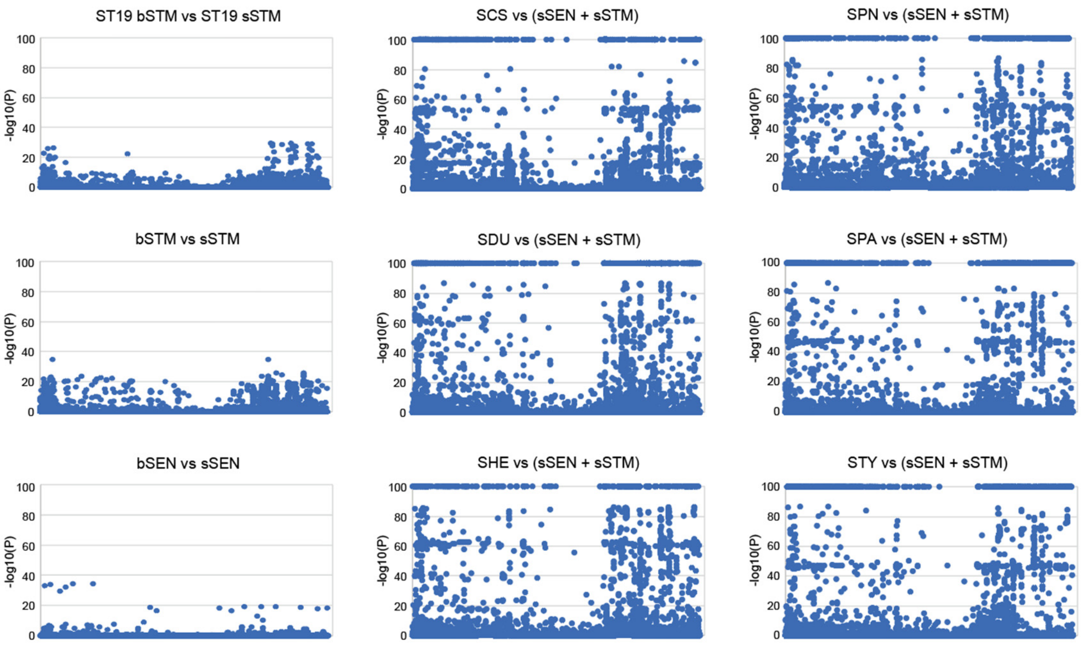

Figure S1. Manhattan plot showing the association of all genetic features with invasiveness. Different sets of *Salmonella* isolates are compared to test whether the features are enriched in the invasive isolates. The p value is calculated by Chi-square test and adjusted by BH method. bSTM, blood-sourced *Salmonella* Typhimurium; sSTM, stool-sourced *Salmonella* Typhimurium; bSEN, blood-sourced *Salmonella* Enteritidis; sSEN, stool-sourced *Salmonella* Enteritidis; SCS, *Salmonella* Choleraesuis; SDU, *Salmonella* Dublin; SHE, *Salmonella* Heidelberg; SPN, *Salmonella* Panama; SPA, *Salmonella* Paratyphi A; STY, *Salmonella* Typhi.

Table S1. Genetic features enriched in invasive *Salmonella* serovars.

Features	Enriched in	Category	Annotation	Positive ratio in				
				sSTMs+sSEN	SCS	SDU	SHE	SPN
pseudo-operon928	SCS SDU SHE SPN	pseudo-operon	hypothetical proteins (STM14_RS21840,STM14_RS25665)	0.0195	0.9947	0.9987	0.9974	0.9985
STM14_RS04335	SCS SDU SHE SPN	pseudogene	hypothetical protein	0.0156	0.9894	0.9987	0.9974	0.9985
SED_RS03370	SCS SDU SHE	CDS	phage protein NinB	0.1306	0.9153	0.8363	0.6848	0.1942
pseudo-operon65	SDU SHE SPN	pseudo-operon	hypothetical protein (STM14_RS02065)	0.3450	0.9947	0.9987	0.9974	0.9985
pseudo-operon139	SDU SHE SPN	pseudo-operon	dpiA,B (STM14_RS24500,STM14_RS03735,STM14_RS03730)	0.3333	0.0053	0.9987	0.9974	0.9985
pseudo-operon163	SDU SHE SPN	pseudo-operon	cytochrome bd-I oxidase (STM14_RS04320-STM14_RS04340)	0.3411	0.9947	0.9987	0.9974	0.9985
pseudo-operon237	SDU SHE SPN	pseudo-operon	hypothetical proteins (STM14_RS05760,STM14_RS05765)	0.3197	0.0159	0.9962	0.9974	0.9970
pseudo-operon378	SDU SHE SPN	pseudo-operon	hypothetical proteins (STM14_RS09385,STM14_RS09390)	0.3294	0.0053	0.9987	0.9974	0.9985
pseudo-operon580	SDU SHE SPN	pseudo-operon	phage proteins (STM14_RS14290-STM14_RS14300)	0.4133	0.9947	0.9987	0.9961	0.9742
pseudo-operon854	SDU SHE SPN	pseudo-operon	membrane proteins (STM14_RS20305,STM14_RS20310)	0.3294	0.9947	0.9987	0.9974	0.9985
pseudo-operon873	SDU SHE SPN	pseudo-operon	adenylate cyclase (STM14_RS20760,STM14_RS20765)	0.3236	0.9947	0.9987	0.9974	0.9985
pseudo-operon874	SDU SHE SPN	pseudo-operon	membrane proteins (STM14_RS20770,STM14_RS20775)	0.3236	0.9947	0.9987	0.9974	0.9985
pseudo-operon876	SDU SHE SPN	pseudo-operon	membrane proteins (STM14_RS20825-STM14_RS20835)	0.3236	0.9947	0.9987	0.9974	0.9985
pseudo-operon937	SDU SHE SPN	pseudo-operon	phage proteins (STM14_RS22050-STM14_RS22075)	0.3314	0.9947	0.9987	0.9974	0.9985
pseudo-operon984	SDU SHE SPN	pseudo-operon	glucuronate isomerase (STM14_RS23190,STM14_RS23195)	0.3333	0.9947	0.9987	0.9624	0.9985
pseudo-operon985	SDU SHE SPN	pseudo-operon	transcriptional regulator (STM14_RS23200)	0.3236	0.9947	0.9987	0.9598	0.9985
pseudo-operon986	SDU SHE SPN	pseudo-operon	dehydratase (STM14_RS23205,STM14_RS23210)	0.3236	0.9947	0.9987	0.9598	0.9985
pseudo-operon987	SDU SHE SPN	pseudo-operon	glucosyl ceramidase (STM14_RS23215,STM14_RS23220)	0.3236	0.9947	0.9987	0.9598	0.9985
pseudo-operon988	SDU SHE SPN	pseudo-operon	dehydrogenase (STM14_RS23240)	0.3255	0.9947	0.9987	0.9598	0.9985
pseudo-operon999	SDU SHE SPN	pseudo-operon	Lon protease (STM14_RS23530-STM14_RS23550)	0.3567	0.9947	0.9987	0.9974	0.9985
pseudo-operon1000	SDU SHE SPN	pseudo-operon	DNA repair ATPase (STM14_RS23555-STM14_RS23565)	0.3275	0.9947	0.9987	0.9974	0.9985
STM14_RS24500	SDU SHE SPN	pseudogene	hypothetical protein	0.3314	0.0000	0.9987	0.9961	0.9985
STM14_RS25980	SDU SHE SPN	pseudogene	hypothetical protein	0.3255	0.9947	0.9974	0.9974	0.9666
STM14_RS09390	SDU SHE SPN	pseudogene	hypothetical protein	0.3255	0.0053	0.9974	0.9974	0.9985

STM14_RS10560	SDU SHE SPN	pseudogene	hypothetical protein	0.4464	0.9947	0.9987	0.9987	0.9985
STM14_RS26235	SDU SHE SPN	pseudogene	hypothetical protein	0.3236	0.0000	0.9987	0.9974	0.9985
SPAB_RS21705	SDU SHE SPN	CDS	hypothetical protein	0.3255	0.9947	0.9962	0.9948	0.9985
STBHUCCB_RS14210	SDU SHE SPN	CDS	transposase	0.1131	0.4709	0.9898	0.9767	0.9939
T_RS05005	SDU SHE SPN	CDS	membrane protein	0.3236	0.0000	0.9450	0.8664	0.9029
pseudo-operon7	SCS SHE SPN	pseudo-operon	sulfatase maturase (STM14_RS00750,STM14_RS00755)	0.0058	0.9947	0.0000	0.9974	0.9985
pseudo-operon372	SCS SHE SPN	pseudo-operon	secretion translocator (STM14_RS09225,STM14_RS09230)	0.0078	0.7619	0.1202	0.9598	0.9985
pseudo-operon963	SCS SHE SPN	pseudo-operon	transcriptional regulator (STM14_RS22655,STM14_RS22660)	0.0000	0.9947	0.0013	0.9974	0.9985
AT698_RS17220	SCS SHE SPN	CDS	hypothetical protein	0.0000	0.9894	0.0000	0.9507	0.9256
NCTC10384_02803	SCS SHE SPN	CDS	hypothetical protein	0.0000	0.9841	0.0000	0.9896	0.9514
SCH_RS00165	SCS SHE SPN	CDS	membrane protein	0.0000	0.9788	0.0000	0.9300	0.9605
SCH_RS00185	SCS SHE SPN	CDS	aryl sulfotransferase	0.0000	0.9947	0.0000	0.9974	0.9985
SCH_RS21890	SCS SHE SPN	CDS	putative periplasmic protein	0.0000	0.9735	0.0000	0.7808	0.8240
SESA_RS02205	SCS SHE SPN	CDS	hypothetical protein	0.0000	0.9947	0.0000	0.9961	0.9863
T_RS12930	SCS SHE SPN	CDS	TioA protein	0.0000	0.9735	0.0000	0.7471	0.7314
T_RS12935	SCS SHE SPN	CDS	transcriptional regulator	0.0000	0.9630	0.0000	0.7043	0.7071
T_RS12940	SCS SHE SPN	CDS	putative fimbrial protein TcfD	0.0000	0.9894	0.0000	0.9909	0.9666
T_RS12945	SCS SHE SPN	CDS	fimbrial outer membrane usher protein TcfC	0.0000	0.9894	0.0000	0.9883	0.9803
T_RS12950	SCS SHE SPN	CDS	putative fimbrial subunit TcfB	0.0000	0.9947	0.0000	0.9961	0.9863
T_RS12955	SCS SHE SPN	CDS	putative fimbrial protein TcfA	0.0000	0.9841	0.0000	0.9922	0.9803
T_RS12960	SCS SHE SPN	CDS	transposase	0.0039	0.9947	0.0000	0.9974	0.9863
pseudo-operon675	SCS SDU SPN	pseudo-operon	sugar dehydrogenase (STM14_RS16460-STM14_RS16475)	0.0234	0.9947	0.9923	0.0039	0.9985

Note: sSTM, stool-sourced *Salmonella* Typhimurium; sSEN, stool-sourced *Salmonella* Enteritidis; SCS, *Salmonella* Choleraesuis; SDU, *Salmonella* Dublin; SHE, *Salmonella* Heidelberg; SPN, *Salmonella* Panama.
